# Copolymer-1 Promotes Neurogenesis and Improves Functional Recovery after Acute Ischemic Stroke in Rats

**DOI:** 10.1371/journal.pone.0121854

**Published:** 2015-03-30

**Authors:** Yolanda Cruz, Jonathan Lorea, Humberto Mestre, Jennifer Hyuna Kim-Lee, Judith Herrera, Raúl Mellado, Vanesa Gálvez, Leopoldo Cuellar, Carolina Musri, Antonio Ibarra

**Affiliations:** 1 Facultad de Ciencias de la Salud, Universidad Anáhuac México Norte, Huixquilucan, Estado de México, México; 2 Centro de Investigación del Proyecto CAMINA A.C. Distrito Federal, México; Fraunhofer Institute for Cell Therapy and Immunology, GERMANY

## Abstract

Stroke triggers a systemic inflammatory response that exacerbates the initial injury. Immunizing with peptides derived from CNS proteins can stimulate protective autoimmunity (PA). The most renowned of these peptides is copolymer-1 (Cop-1) also known as glatiramer acetate. This peptide has been approved for use in the treatment of multiple sclerosis. Cop-1-specific T cells cross the blood-brain barrier and secrete neurotrophins and anti-inflammatory cytokines that could stimulate proliferation of neural precursor cells and recruit them to the injury site; making it an ideal therapy for acute ischemic stroke. The aim of this work was to evaluate the effect of Cop-1 on neurogenesis and neurological recovery during the acute phase (7 days) and the chronic phase of stroke (60 days) in a rat model of transient middle cerebral artery occlusion (tMCAo). BDNF and NT-3 were quantified and infarct volumes were measured. We demonstrated that Cop-1 improves neurological deficit, enhances neurogenesis (at 7 and 60 days) in the SVZ, SGZ, and cerebral cortex through an increase in NT-3 production. It also decreased infarct volume even at the chronic phase of tMCAo. The present manuscript fortifies the support for the use of Cop-1 in acute ischemic stroke.

## Introduction

Acute ischemic stroke is a leading cause of serious long-term disability in adults [1]. Current treatment options are directed towards arterial recanalization using fibrinolytic agents such as tissue plasminogen activator (tPA). However, central nervous system (CNS) damage is known to trigger a systemic inflammatory response that may exacerbate brain injury [2]. Several studies have demonstrated that the modulation of this inflammatory response results in protective autoimmunity (PA)[3–5]. PA has shown to improve functional recovery in animal models of stroke, spinal cord injury, Parkinson, and Alzheimer disease[6–9]. Immunizing with peptides derived from proteins found in the CNS can stimulate PA. Perhaps the most renown of these peptides is copolymer-1 (Cop-1) also known as glatiramer acetate (commercialized by Teva Pharmaceuticals under Copaxone). Cop-1 is a synthetic peptide that consists of four amino acids (alanine, lysine, glutamic acid, and tyrosine) in a fixed molar ratio[10]. This peptide has been approved for use in the treatment of relapsing-remitting multiple sclerosis (MS) after many studies demonstrated its ameliorating effect on experimental allergic encephalomyelitis (EAE; animal model of MS) [11,12]. Subcutaneous immunization with Cop-1 has pleiotropic properties, among them are: i) an inhibitory effect on monocyte reactivity limiting their production of tumor necrosis factor α (TNFα) and interleukin-12 (IL-12), and increasing IL-10 and transforming growth factor β (TGFβ); ii) activation of Th2/3 and regulatory T cells (Treg); iii) Cop-1-specific Th2/3 cells cross the blood-brain barrier and secrete neurotrophins and anti-inflammatory cytokines; iv) elevated proliferation of neural precursor cells (NPC), proliferation and recruitment into the injury site [13]. The myriad processes influenced by PA and immunization with Cop-1 indicate that it is an ideal therapy for acute ischemic stroke. This strategy has already shown to improve neurological recovery and decrease infarct volume 7 days after stroke in a rat model of transient middle cerebral artery occlusion (tMCAo) [4]. Although, it has recently been suggested that there is no benefit to using this therapy [14,15].

The adult brain has the capacity for self-repair after insults[16]. This process known as neurogenesis is the ability to generate functional neurons from neural stem cells[17]. These new neurons are continuously generated in two regions: the subventricular zone (SVZ), lining the lateral ventricles and the subgranular zone (SGZ) of the dentate gyrus in the hippocampus [17]. After stroke, neuroblasts—expressing doublecortin (Dcx)—are capable of travelling from the SGZ and SVZ towards the infarct[16]. More importantly, these neuroblasts can differentiate into functional neurons in the striatum and cerebral cortex [18]. This process is susceptible to many molecular cues [17]. Neurogenesis is enhanced by brain-derived neurotrophic factor (BDNF), neurotrophin-3 (NT-3), IL-4, and TGFβ [19,20]. Furthermore, it can also be inhibited by TNFα and interferon γ (IFNγ) [21]. Treatment with Cop-1 elevates pro-neurogenic molecules (i.e. BDNF, NT-3 and IL-4) while diminishing TNFα, IL-1β, and IFNγ [9,12,22]. These properties make Cop-1 an ideal candidate for the treatment of acute ischemic stroke.

Our previous study observed a substantial benefit to treatment with Cop-1 after tMCAo in rats [4]. However, two separate studies have suggested that GA (Copaxone) is ineffective in treating murine models of MCAo [14,15]. This discrepancy in findings may be due to short follow-up period duration. Maximum cell proliferation in the SVZ occurs at 1 to 2 weeks after tMCAo [16]. More recent studies have even found an increased number of neurospheres 6 weeks after stroke [18]. Data suggests that studies with follow-ups of a week (7 days) or shorter will probably not be able to observe the increase in neurogenesis and the resultant functional improvement due to immunization with Cop-1.

The aim of this study was to evaluate the effect of Cop-1 on neurogenesis and neurological recovery during the acute phase (7 days) and the chronic phase of stroke (60 days) in a rat model of tMCAo. We also quantified neurotrophin concentration at the acute time point to find out which neurotrophin mediates Cop-1-induced neurogenesis. At the end of the two-month follow-up period we quantified infarct volumes to see if neuroprotection and neurogenesis had any effect on the size of the necrotic core.

## Materials and Methods

### Ethical statement

All experiments were performed according to the National Institutes of Health Guide for the Care and Use of Laboratory Animals, the Mexican Official Norm of Principles for the Care of Laboratory Animals. The study was approved by the Institutional Animal Care and Use Committee and Institutional Review Board of Universidad Anahuac Mexico Norte (ID: CSCBIAAAIJ080810086). All experiments were designed and reported according to the ARRIVE guidelines (S1 ARRIVE Guidelines Checklist) [23].

### Experimental animals

Sample size calculation using our previous study (α: 0.05; β: 0.90) yielded a sample size of 70 animals for all necessary experiments taking into account postsurgical mortality and chronic complications of stroke[4,24]. The Animal Breeding Center of CAMINA Research Project provided 70 male Sprague-Dawley rats (335 ± 15 g.; 9 ± 1 weeks old). The rats were housed in individually ventilated temperature and humidity-controlled cages (age-matched two per cage) with hardwood chip bedding in a 12 hr. light/dark cycle room. All animals had free access to food and water.

### Study design

**Fig 1:**
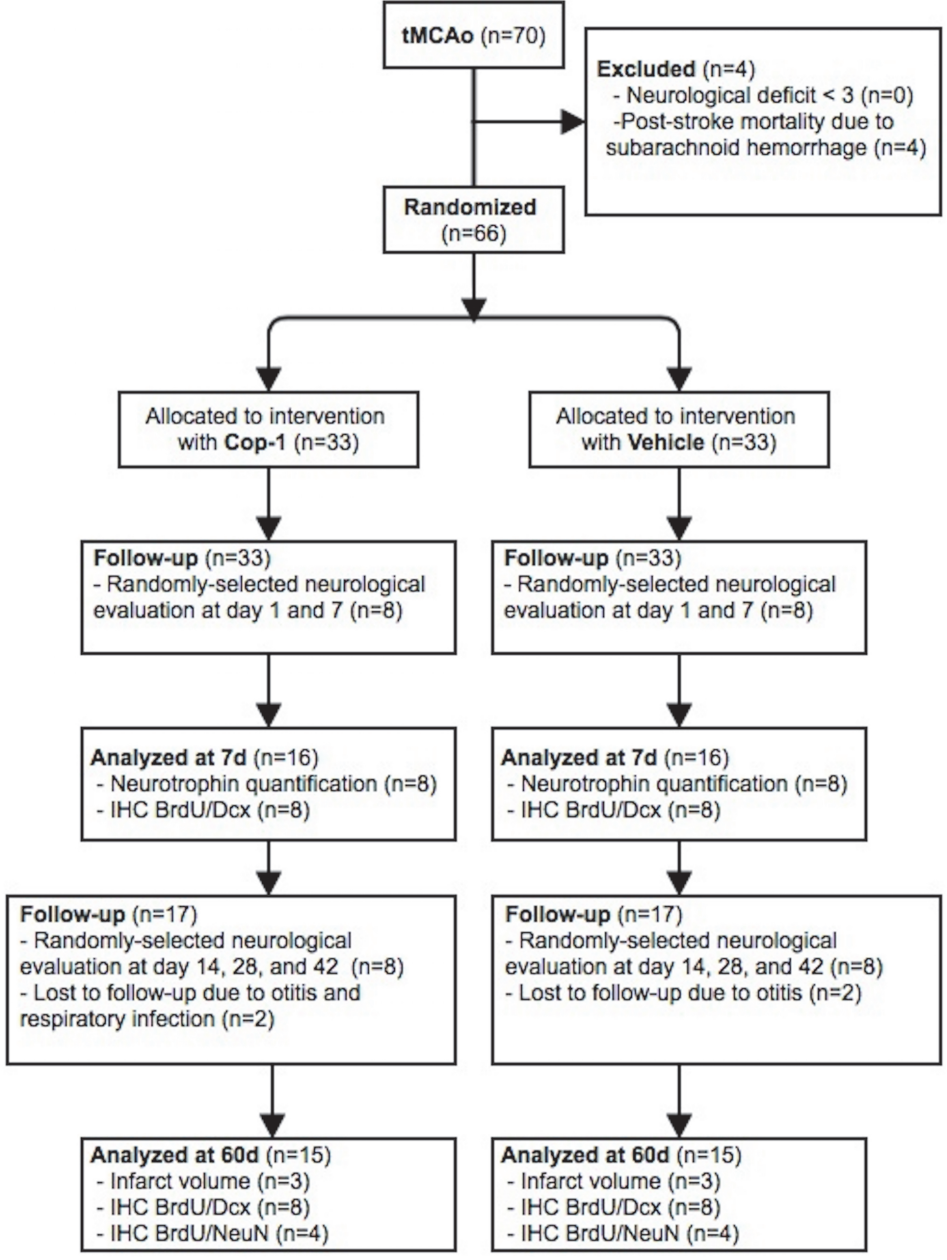
Study design flow chart and time schedule. IHC: immunohistochemistry; BrdU: 5-bromo-2’-deoxyuridine; Dcx: doublecortin. Animals lost to follow-up due to otitis or respiratory infection were humanely euthanized by CO_2_ inhalation.

Prior to tMCAo surgery, all animals were randomly allocated (GraphPad QuickCalcs: http://www.graphpad.com/quickcalcs/) to either intervention with Cop-1 or vehicle by a third party. After baseline statistical analysis for weight and age yielded no significant difference between groups the allocation key was masked. An investigator not in contact with the experimental animals prepared the immunizations and labeled each syringe with a masked code. The physical appearance of the syringe content was identical and could not be differentiated. Surgeons were only handed a list with subject number and group; therefore blinded to the allocation group before the procedure. To analyze neurological recovery animals were randomly selected (*n =* 8) and evaluated by three experts blinded to the allocation group. Animals that did not achieve a neurological deficit greater than 3 (see below) 30 minutes after tMCAo were excluded from the study (*n =* 0). Four animals were lost to post-operative mortality (subarachnoid hemorrhage). At day 7, 32 rats were used for neurotrophin quantification (*n =* 8 per group) and neurogenesis analysis (*n =* 8 per group). Throughout the study 4 animals were lost to ear and respiratory tract infections. Upon termination of the follow-up period (60 days), 32 rats were used for neurogenesis analysis (*n =* 8 per group), evaluation of newly formed neurons (*n =* 4 per group), and infarct volume quantification (*n =* 4 per group). All experiments were done once. The study design is presented as a flow chart in Fig 1.

### Transient middle cerebral artery occlusion (tMCAo)

The procedure was performed using the transient middle cerebral artery occlusion (tMCAo) model previously reported [25]. Anesthesia was induced with 4% isoflurane (Lisorane, Baxter, Puerto Rico) and a maintenance dose of 1.5% was used throughout the procedure. Body temperature was maintained at 37°C with a heating pad during surgery and afterwards until recovery from anesthesia. The left common carotid artery (CCA), external carotid artery (ECA), and internal carotid artery (ICA) were exposed through a midline incision. The ECA was ligated, cauterized, and cut. The ICA was then isolated to avoid damage to the vagal nerve. A 3–0 monofilament nylon suture (Ethicon, Johnson and Johnson, Mexico City, Mexico) with a flame-rounded head was inserted through the ICA via a small incision in the ECA stump. The distance from bifurcation of the CCA to the tip of the suture was approximately 18 mm in all rats, consistent with published descriptions of the tMCAo model. After 90 min of occlusion the suture was withdrawn and cerebral blood flow restored. The skin was sutured and the rats were allowed to recover. All animals received paracetamol (200 mg/kg; twice a day; PO; Tempra) and enrofloxacine (10mg/kg; once a day; SC; Baytril) for three days after surgery.

### Immunizations

Animals were injected with 200 μg of Cop-1 (Sigma, St. Louis, USA) dissolved in saline solution (SS) and emulsified in an equal volume of complete Freund’s adjuvant (CFA) containing 5 mg/ml of *Mycobacterium tuberculosis* H37 RA (Sigma, St. Louis, USA) in a total volume of 150 μl. Control animals received the same volume of SS and CFA. All immunizations were applied subcutaneously at the interscapular space immediately after reperfusion. The only adverse event observed after immunization was erythema at the injection site in 6/70 animals.

### Neurological deficit evaluation

Animals were tested for neurological deficits on day 1, 7, 14, 28, 42, and 60. The evaluation was performed using the following neurological scale: Briefly, 0, no deficit; 1, failure to fully extend the contralateral forepaw; 2, circling towards the contralateral side; 3, falling to the left; 4, no spontaneous walking and/or a depressed level of consciousness[25].

### Enzyme-linked immunosorbant assay (ELISA)

After lethal pentobarbital injection, rats were craneotomized and brain samples were rapidly excised. The tissue samples were weighed and snap frozen in liquid nitrogen prior to storage at -70°C. Within two weeks of freezing, tissue samples were homogenized in ice cold homogenization buffer consisting of 100 mM Tris/Hcl, pH 7, 2% bovine serum albumin (BSA), 1 M NaCl, 4mM EDTA, 2% Triton X-100, 0.1% NaN_3_, and the following protease inhibitors: 5 μg/mL aprotinin, 0.5 μg/mL antipain, 157 μg/mL benzamidine, 0.1 μg/mL pepstatin A and 17 μg/mL phenylmethyl-sulphonyl fluoride. Homogenates were prepared in approximately 20 volumes of the homogenization buffer to tissue-wet weight. The homogenates were centrifuged at 14,000xg for 30 minutes. The resulting supernatants were divided into two equal samples and used for the BDNF and NT-3 assay. The samples were analyzed by triplicate and following the instructions of the ChemiKine BDNF and NT-3 Sandwich ELISA Kit (Millipore, USA). Absorbance was measured in a microplate spectrophotometer at a 450nm wavelength (MultiSkan, Thermo Scientific, Finland).

### Immunohistochemistry

Neurogenesis was evaluated by way of immunofluorescence using a double stain with 5-bromo-2’-deoxyuridine (BrdU) and doublecortin (Dcx). BrdU is incorporated into metabolically active post mitotic cells and Dcx is a marker for neural precursor cells (NPC), so BrdU^+^/Dcx^+^ cells are a result of neurogenesis. Animals were evaluated at 7 days and 2 months after tMCAo. At 24 hours and 2 hours prior to euthanasia, animals were injected with BrdU (Roche, 50 mg/kg; IP). To evaluate if an increase in neurogenesis resulted in mature neurons a double stain for BrdU and NeuN was used in the ipsilateral cerebral cortex at 60 days. For this assay animals were injected with BrdU on day 28 and 29 as well as 24 h and 2 h prior to sacrifice. Brains were procured by paraformaldehyde perfusion as previously mentioned[4]. Coronal cuts of SVZ, DG and cortex were obtained by cryosectioning (three slices 40 μm-thick with 200 μm spacing, a total of 9 sections per animal were counted) and placed on slides using the free float method. Slides were washed twice for 10 min with phosphate-buffered saline with Triton (PBT) and incubated with ImmunoRetriever for 30 min at 65°C. Afterwards, they were subjected to two washes of 5 min with phosphate-buffered saline (PBS) and then again incubated with 1N HCl at 37°C. When completed they were incubated for 10 min with sodium borate 0.1 M and again washed three times with PBT. Unspecific binding sites were blocked with standard blocking solution for 30 min. The primary antibodies against BrdU (Roche, mouse IgG, 1:250), Dcx (Santa Cruz, goat IgG, 1:250), and NeuN (Millipore, mouse IgG, 1:250) were allowed to incubate for 16 h overnight. Next day, slides were washed three times for 10 min with PBT and the secondary antibodies (BrdU: donkey IgG; Dcx: rabbit IgG; NeuN: donkey; all at 1:500 from Invitrogen) were incubated for 2 hours. Excess antibodies were removed by washing with PBT. Slides were counterstained with DAPI. All areas were quantified as total number of cells in the ipsilateral and contralateral hemisphere by a blinded evaluator using cell counting software (Image-Pro Plus, Media Cybernetics, USA). The total number of BrdU^+^/Dcx^+^ cells was obtained by averaging the total number of cells from all 9 slides. Photomicrographs were taken in an epifluorescence microscope (Olympus BX41) with an Olympus DP72 camera.

### Infarct volume measurement

To analyze the size of infarct area, rats were anesthetized 60 days after ischemia, and perfused via the ascending aorta, with 50 ml NaCl 0.9% saline, followed by 500 ml 4% paraformaldehyde, using a peristaltic pump at 30 ml/min. The brains were removed, placed in the same fixing solution for 24 h and then cryoprotected in a 30% sucrose solution for at least 3 days. Three coronal cryosections 25-μm thick were cut every 200 μm and collected onto gelatin-coated slides. To determine the area of lesion, 30 evenly spaced sections of the brain were observed by a pathologist blinded to the treatment group after staining them with hematoxylin–eosin. A computer image analysis system (Image-Pro Plus, Media Cybernetics, USA) was used for the evaluation. Infarct area in each slice was calculated as follows: measured infarct area × (total contralateral hemisphere area/total ipsilateral hemisphere area). Infarct volumes of each rat were then computed by integrating infarct areas of sequential brain sections. Total ischemic volume in the ipsilateral hemisphere was determined as a percentage of the volume of the contralateral (control) hemisphere.

### Statistical methods

Statistical analysis was done using the Prism 6 software (Prism 6.0, GraphPad Software Inc. San Diego, USA). Data is expressed as mean ± standard deviation (SD). All data sets were analyzed for normality using the D’Agostino & Pearson omnibus K2 normality test. If data passed the normality test, parametric two-tailed unpaired t tests were used; if not two-tailed Mann-Whitney U tests were employed. Neurological recovery throughout the follow-up period was analyzed using two-way repeated measures ANOVA with Sidak’s multiple comparisons test. Correlation between neurogenesis and functional recovery was done using Pearson’s correlation coefficient. All exact P values less than 0.05 were considered statistically significant.

## Results

### Immunization with Cop-1 improves neurological recovery after tMCAo

**Fig 2:**
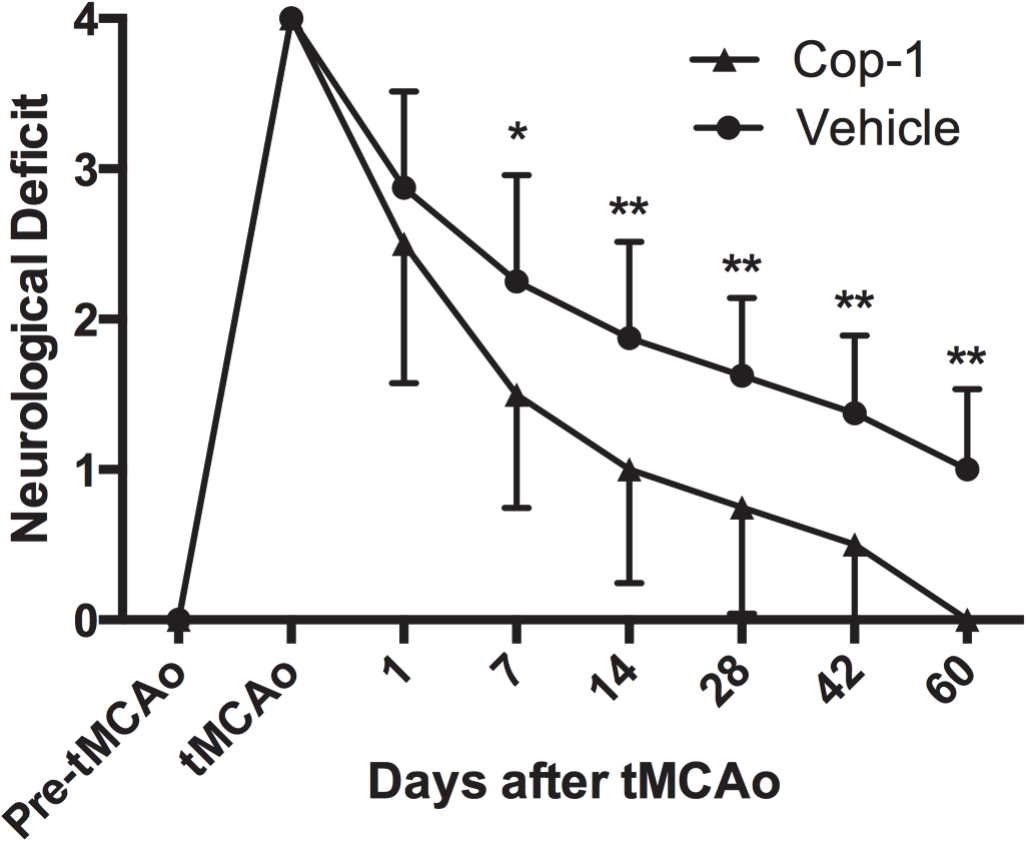
Neurological recovery after immunization with Cop-1. The effect of treatment with Cop-1 on neurological deficit after tMCAo. A lower score implies a better clinical recovery. Animals were randomly selected from the cohort (*n =* 8 per group) for evaluation at day 1, 7, 14, 28, 42, and 60 by three experts blinded to the group allocation. The animals treated with Cop-1 had a statistically significant recovery starting from day 7 and up to day 60. Data was analyzed with a two-way repeated measure ANOVA and Sidak’s post hoc multiple comparisons test. * *P <* 0.05; ** *P <* 0.01. tMCAo: transient middle cerebral artery occlusion.

The day before the surgical procedure all animals (*n =* 70) were tested for neurological deficit, baseline data demonstrates that all subjects had a score of 0. Immediately after tMCAo, neurological evaluation showed that all animals had a score of 4. At day 1, there was no significant difference between Cop-1 and vehicle-treated animals (Fig 2: 2.5 ± 0.9 vs. 2.9 ± 0.6 respectively; *P >* 0.05). At day 7 after tMCAo the difference between the experimental group and the control begins to become apparent and statistically significant (1.5 ± 0.8 *vs*. 2.3 ± 0.7; *P <* 0.05). These results are in line with our previous study [4]. The accelerated recovery of animals that received immunization with Cop-1 against vehicle becomes more pronounced at day 14 (1.0 ± 0.8 *vs*. 1.9 ± 0.6; *P <* 0.01), 28 (0.8 ± 0.7 *vs*. 1.6 ± 0.5; *P <* 0.01), 42 (0.5 ± 0.5 *vs*. 1.4 ± 0.5; *P <* 0.01), and 60 (0.0 ± 0.0 *vs*. 1.0 ± 0.5; *P <* 0.01).

### Cop-1 increases neurogenesis in the SVZ, SGZ, and the ipsilateral cerebral cortex in the acute phase of tMCAo

**Fig 3:**
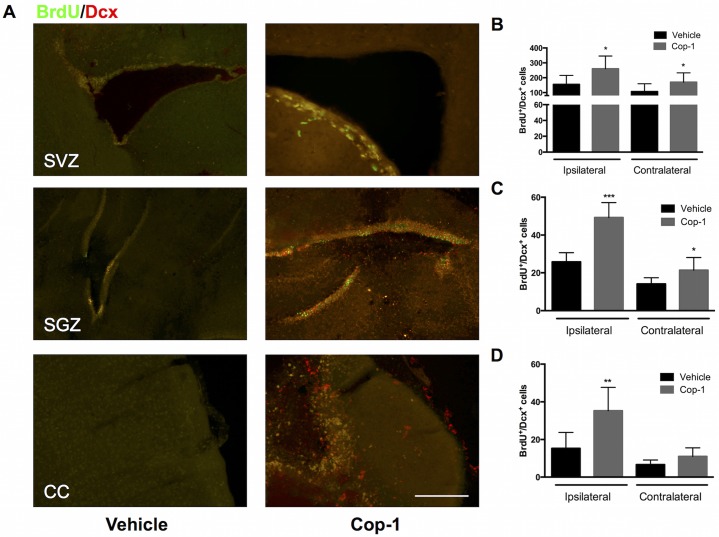
Neurogenesis in the SVZ, SGZ, and CC 7 days after tMCAo. The effect of immunizing with Cop-1 on neurogenesis in the SVZ, SGZ, and CC of the ipsilateral and contralateral hemispheres. (A) Merged photomicrographs represent double staining for BrdU and Dcx at the SVZ, SGZ, and CC of the ipsilateral hemisphere; an evaluator blinded to the group allocation counted the number of BrdU+/Dcx+ cells in all slides. 10x magnification, scale bar: 100 μm. (B) Number of BrdU^+^/Dcx^+^ cells in the ipsilateral and contralateral SVZ in Cop-1-treated and vehicle-treated rats. (C) Number of BrdU^+^/Dcx^+^ cells in the ipsilateral and contralateral SGZ in Cop-1-treated and vehicle-treated rats. (D) Number of BrdU^+^/Dcx^+^ cells in the ipsilateral and contralateral CC in Cop-1-treated and vehicle-treated rats. Data was tested for normality and analyzed using a two-tailed Mann-Whitney U test between Cop-1 and vehicle in each region for each hemisphere (*n =* 8 per group). * *P <* 0.05, ** *P <* 0.01, *** *P <* 0.001. SVZ: subventricular zone, SGZ: subgranular zone, CC: cerebral cortex.

To analyze neurogenesis we used BrdU (a thymidine analogue that is incorporated into the DNA of replicating cells) to identify recently post-mitotic cells. We then used Dcx to label NPCs or neuroblasts that could be travelling towards the necrotic core. Cells that co-localized BrdU and Dcx (BrdU^+^/Dcx^+^) were considered NPCs and therefore a sign of active neurogenesis. This process was evaluated at the two known sites for adult neurogenesis: the subventricular zone (SVZ) lining the lateral ventricles and the subgranular zone (SGZ) of the dentate gyrus in the hippocampus. To understand if travelling NPCs were arriving at the necrotic core or penumbra we analyzed the cerebral cortex. At 7 days post-tMCAo we observed that the SVZ presented the most neurogenesis, which is expected (Fig 3A). During this time, Cop-1-treated animals exhibited significantly more neurogenesis in the ipsilateral SVZ (Fig 3B, Cop-1 *vs*. Vehicle: 260 ± 86 *vs*. 155 ± 61; *P <* 0.05) and contralateral SVZ (Fig 3B, 170 ± 63 vs. 107 ± 53; P < 0.05). The SGZ showed less neurogenesis than the SVZ but immunized animals had significantly more BrdU^+^/Dcx^+^ cells than controls in the ipsilateral SGZ (Fig 3C, 49.3 ± 7.9 *vs*. 25.8 ± 4.9; *P <* 0.001) and contralateral SGZ (Fig 3C, 21.4 ± 6.7 *vs*. 14.1 ± 3.2; *P <* 0.05). More interestingly, we found a greater presence of travelling NPCs at the ipsilateral cerebral cortex—especially in the peri-infarct region of Cop-1-treated subjects (Fig 3D, 35.3 ± 12 *vs*. 15.3 ± 8.5; *P <* 0.01). No difference was observed in the contralateral hemisphere meaning that the NPCs were actively migrating towards the ischemia-damaged zone (Fig 3D). At 7 days, the neurological deficit did not correlate with neurogenesis for any of the regions.

### Increased neurogenesis after treatment with Cop-1 is mediated, in part, by NT-3 but not BDNF

**Fig 4:**
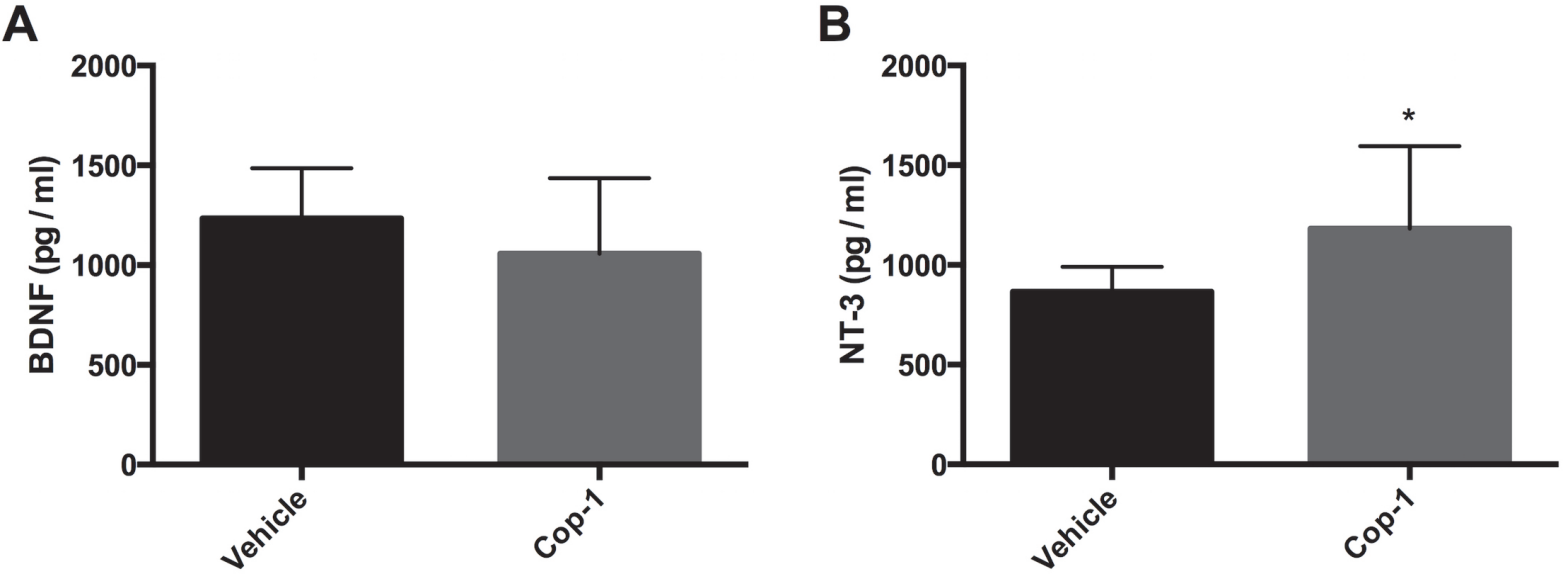
BDNF and NT-3 production 7 days after tMCAo. Neurotrophin quantification at the ischemic zone in Cop-1-treated and vehicle animals (*n =* 8 per group). ELISA analysis established that NT-3 production is elevated in rats that received Cop-1 immunization but not so for BDNF. (A) Concentration of BDNF and (B) NT-3 in the ipsilateral hemisphere. Data was tested for normality and analyzed using a non-parametric Mann-Whitney U test (*n =* 8 per group). * *P <* 0.05. pg/mL: picograms per milliliter.

In order to elucidate which neurotrophin could be responsible for enhancing neurogenesis after Cop-1 immunization we decided to quantify these within the ischemic zone 7 days post-tMCAo. Of the neurotrophin family, some of the most implicated in neurogenesis are BDNF and NT-3. ELISA analysis of infarct samples revealed an unexpected result. Previous studies have demonstrated that GA increases the production of BDNF *in situ* [12]. Our experiments showed that BDNF concentration 7 days after stroke did not vary between Cop-1 and vehicle groups (Fig 4A, 1057 ± 378 *vs*. 1234 ± 252 pg/mL respectively; *P >* 0.05). Moreover, we were able to demonstrate that the quantity of NT-3 within the infarct was significantly higher in Cop-1-treated animals (Fig 4B, 1182 ± 414 *vs*. 865 ± 125 pg/mL; *P <* 0.05). This data suggests that the increased neurogenesis seen in the Cop-1 cohort during the acute phase after tMCAo can be mediated—at least to a certain extent—by an increased production of NT-3.

### Immunizing with Cop-1 enhances neurogenesis in the SVZ, SGZ, and CC even in the chronic phases after tMCAo

**Fig 5:**
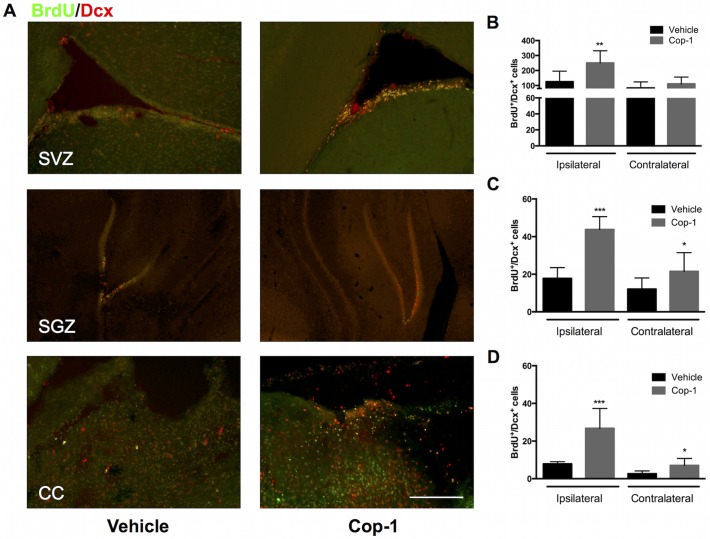
Neurogenesis in the SVZ, SGZ, and CC 60 days after tMCAo. The effect of immunizing with Cop-1 on neurogenesis in the SVZ, SGZ, and CC of the ipsilateral and contralateral hemispheres. (A) Merged photomicrographs represent double staining for BrdU and Dcx at the SVZ, SGZ, and CC of the ipsilateral hemisphere; an evaluator blinded to the group allocation counted the number of BrdU+/Dcx+ cells in all slides. 10x magnification, scale bar: 100 μm. (B) Number of BrdU^+^/Dcx^+^ cells in the ipsilateral and contralateral SVZ in Cop-1-treated and vehicle-treated rats. (C) Number of BrdU^+^/Dcx^+^ cells in the ipsilateral and contralateral SGZ in Cop-1-treated and vehicle-treated rats. (D) Number of BrdU^+^/Dcx^+^ cells in the ipsilateral and contralateral CC in Cop-1-treated and vehicle-treated rats. Data was tested for normality and analyzed using a two-tailed Mann-Whitney U test between Cop-1 and vehicle in each region for each hemisphere (*n =* 8 per group). * *P <* 0.05, ** *P <* 0.01, *** *P <* 0.001.

In order to justify the marked clinical improvement seen in treated animals we analyzed neurogenesis after 60 days. Cop-1-immunized subjects presented increased levels of neurogenesis in all regions evaluated (Fig 5A). Again, the SVZ had the highest numbers of BrdU^+^/Dcx^+^ cells; however, these were only significantly elevated in the ipsilateral SVZ of animals that received Cop-1 (Fig 5B, Cop-1 *vs*. Vehicle: 250 ± 81 *vs*. 125 ± 70 cells; *P <* 0.01). This effect was no longer witnessed in the SVZ of the contralateral hemisphere (Fig 5B, 111 ± 45 *vs*. 84 ± 40; *P >* 0.05). Enhanced neurogenesis was preserved in the dentate gyrus after Cop-1 treatment in both the ipsilateral SGZ (Fig 5C, 44 ± 6.9 *vs*. 18 ± 5.8; *P <* 0.001) and the contralateral SGZ (Fig 5C, 22 ± 9.9 *vs*. 12 vs. 5.9; *P <* 0.05). The aforementioned regions are known to be active sites of neurogenesis throughout adulthood. However, after tMCAo the most consequential deficit is secondary to the loss of cortical and striatal neurons that receive blood supply from the MCA. Under physiological conditions, there is generally no neurogenesis in the cerebral cortex. Nonetheless, we found a significant amount of BrdU^+^/Dcx^+^ cells in the ipsilateral CC (Fig 5D, 26.8 ± 10.6 *vs*. 7.9 ± 1.1; *P <* 0.001) and contralateral CC (Fig 5D, 7.0 ± 3.8 *vs*. 2.6 ± 1.5; *P <* 0.05). The increase in neurogenesis in all regions correlated highly with the improvement in functional recovery (SVZ: *r =* -0.80, *P =* 0.0002; SGZ: *r =* -0.78, *P =* 0.0004; CC: *r =* -0.67, *P =* 0.004). The relationship between neurological deficit and neurogenesis had a negative correlation due to the fact that the scale used to evaluate deficit defines greater improvement as a lower score. A greater presence of NPCs in the SVZ, SGZ, and CC could be the reason for the clinical recovery observed in treated animals.

### Cop-1 induces the formation of new neurons in the ipsilateral CC and reduces infarct volume in the chronic phases of tMCAo

**Fig 6:**
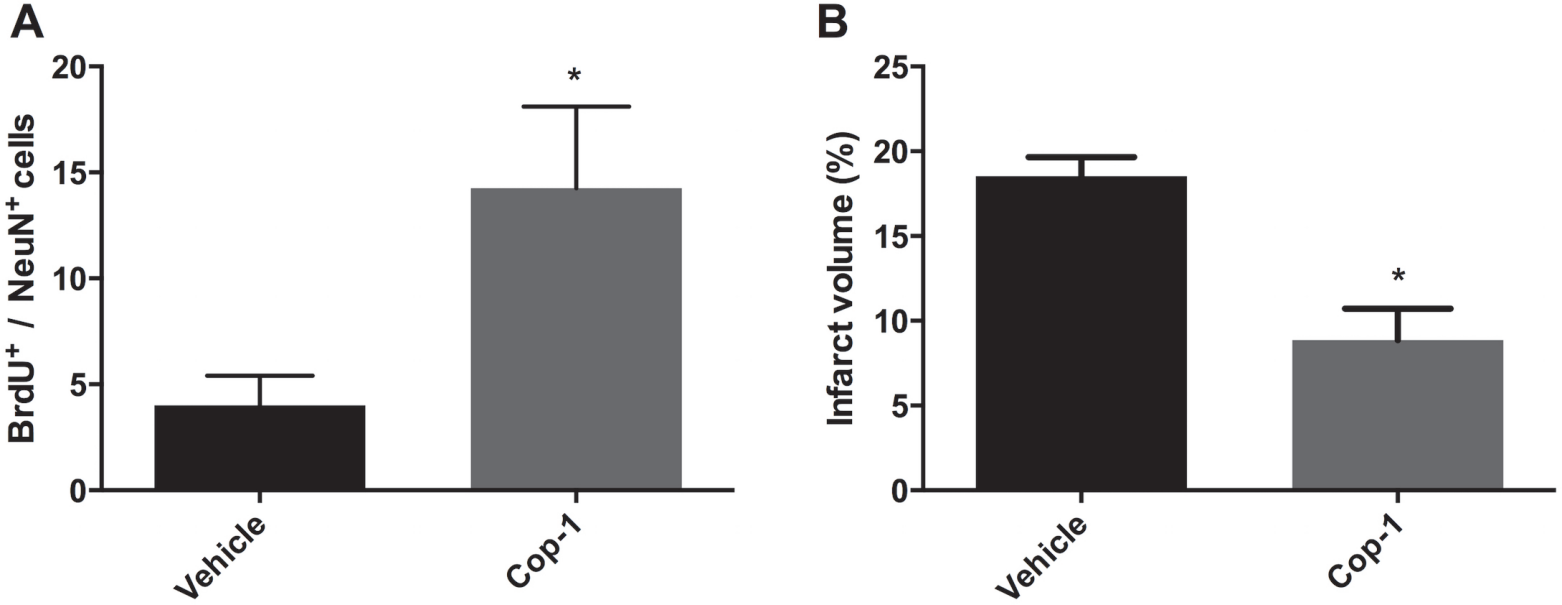
Formation of new neurons and infarct volume analysis 60 days after tMCAo. To better understand if the increase in NPCs was resulting in more new neurons we measured the presence of BrdU^+^/NeuN^+^ cells in the ipsilateral CC. As a measure of tissue protection during the acute phase or tissue regeneration during the chronic phase we also analyzed infarct volume. (A) Quantification of BrdU^+^/NeuN^+^ cells from merged photomicrographs of the peri-infarct region (not shown) at 40x magnification. Two-tailed Mann-Whitney U test (*n =* 4 per group). (B) Infarct volume analysis. Data is expressed as the percentage of the volume of the contralateral hemisphere. Two-tailed Mann-Whitney U test (*n =* 3 per group). * *P <* 0.05.

As a result of the previous experiment we wanted to evaluate if the NPCs migrating to the CC were in fact differentiating into new neurons. To prove this we double labeled BrdU^+^ cells with NeuN, a marker for mature neurons. Cells that co-localized BrdU^+^/NeuN^+^ were considered new neurons; a researcher blinded to the allocation groups counted the cells in all slides. New neurons were quantified within the ipsilateral CC, in the region around the infarct. Treatment with Cop-1 significantly increased the number of new neurons 60 days after tMCAo (Fig 6A Cop-1 vs. Vehicle: 14.3 ± 3.9 *vs*. 4.0 ± 1.4; *P <* 0.05). In an effort to see if the formation of new neurons affected the size of the necrotic core we quantified infarct volume. Volume analysis indicated that animals subjected to treatment with Cop-1 had significantly smaller infarcts than controls (Fig 6B, 8.9 ± 1.9 *vs*. 18.5 ± 1.1%; *P<* 0.05). The profound differences in volume after such a long follow-up suggest that new tissue formed around the infarct making it smaller. On the other hand, it can also be secondary to increased neuroprotection during the acute phase, resulting in less tissue necrosis.

## Discussion

These results further validate the benefits of Cop-1 immunization in the treatment of acute ischemic stroke. Our present study successfully replicated our previous results demonstrating that it improves functional recovery 7 days after tMCAo in a rat model. Moreover, we have added the assertion that Cop-1 increases neurogenesis in the ipsilateral SVZ, SGZ and the CC. This phenomenon was ascribed to an increase in NT-3 production but not BDNF. Th 2/3 lymphocytes activated towards GA have shown to secrete BDNF *in situ*[12]. These cells also have the potential to secrete NT-3, NT-4/5, and other neurotrophins[13]. It appears that our observations contradict *in vitro* evidence of GA-specific T cells. Recent works have found this paradoxical response to Cop-1 *in vivo*. A study working with retinal ganglion cells described that Cop-1 immunization downregulates BDNF while promoting NT-3 expression[5]. The exact effect of *in vivo* Cop-1 immunization on neurotrophin production should be further clarified. The present study corroborated the beneficial effect Cop-1 after tMCAo by demonstrating that it significantly improves neurological deficit 60 days after. A neurogenesis assay showed that treated animals had significantly more BrdU^+^/Dcx^+^ cells in the SVZ, SGZ, and CC at the chronic phases of disease. These cells most likely represent NPCs that are migrating towards the infarct to replenish cortical and striatal neurons. The better recovery observed in these animals correlated with the presence of NPCs. This assumption is further endorsed by the fact that more new neurons (BrdU^+^/NeuN^+^ cells) are present in the peri-ischemic area of Cop-1 immunized rats. GA-reactive T cell clones are known to produce anti-inflammatory cytokine such as IL-4, IL-10 and TGFβ while suppressing pro-inflammatory molecules like IFNγ, TNFα and IL-1β[10]. This modulation of the CNS microenvironment induces a pro-neurogenic state, but also prevents demyelination, augments remyelination, increases proliferation, maturation and survival of oligodendrocyte progenitor cells—as well as other glial cells that aid neuronal survival[13]. This increase in cellular proliferation was also seen in our study when Cop-1-treated animals showed a significant increase in peri-infarct BrdU^+^/Dcx^-^ cells (data not shown). The time points used to assess neurogenesis in the present study were selected taking into consideration the peak of T lymphocyte arrival following cerebral ischemia (7 days) and the phase of stability—a chronic stage—with lower amounts of immune cells [26,27]. At both time points, Cop-1 immunization was capable of inducing a significant increase in neurogenesis. Finally, neuroprotection in the acute phase and/or neuroregeneration in the chronic phase caused a significant shrinkage in the infarct volume of Cop-1-treated subjects. It is important to mention that we had previously demonstrated the beneficial effect of Cop-1 on the outcome of infarct volume [4]. Although the present work was adequately powered, it had smaller sample sizes for the measurement of infarct volume. However, this effect was also seen in our previous pilot study, which had repeated independent experiments and a larger sample size. All the aforementioned data strongly suggests the beneficial effects of Cop-1 in the treatment of acute ischemic stroke but larger multi-center confirmatory preclinical studies are required.

Cop-1 has been used for many years in the treatment of several neurodegenerative diseases. Its success in suppressing EAE earned its approval for the treatment of multiple sclerosis. The fact that Cop-1, or GA (Copaxone), has already undergone phase I clinical trials means that the safety of this drug has been validated. The translation of this study is relatively feasible due to the fact that using GA for acute ischemic stroke would only entitle an off-label use of Copaxone. However, in order to assure a successful translation from the bench to the bedside we must obtain a definite position on the true potential of Cop-1 in stroke therapy. Recent studies have obfuscated this consensus. A recent study evaluated the effect of GA on two murine models of transient and permanent MCAo [14]. Administration of GA did not reduce infarct volume or improve neurological deficit after transient or permanent MCAo. The authors state that significant neurogenesis was found at day 7 but only in the permanent occlusion model. Data also demonstrated that GA-treated animals that suffered a tMCAo produced significantly less microglial pro-inflammatory cytokine (i.e. IL-1β and TNFα). Several issues arise that could explain the contrasting results. The first is the presentation and dose of GA; the previously mentioned study used Copaxone (Teva Pharmaceuticals) at a dose of 67–80 mg/kg. Our model employed the peptide Cop-1 emulsified in an equal volume of CFA at a dose of 0.57–0.63 mg/kg after T cell dose-response proliferation assays yielded this as the optimal concentration (data not shown). It is of interest to mention that we used 100 times less antigen but with a stronger adjuvant and achieved a therapeutic effect. In their discussion, the authors state that they preformed an unsuccessful experiment with GA + CFA and CFA only but this data is not included. Further studies are warranted to define the therapeutic dose of Cop-1/GA and the efficacy of the adjuvants utilized. Poittevin et al. also had a follow-up period of 7 days, which according to our present study is not enough to truly appreciate the beneficial effects of Cop-1 treatment. Another issue raised in the paper is the strain of mice used. The study was done with C57Bl/6 mice that are a Th1-type inbred strain that is susceptible to EAE[28]. After CNS injury, these strains develop an uncontrolled autoreactive response to myelin elements. This idiosyncrasy could nullify C57Bl/6 mice’s response to GA therapy. The true role of EAE- susceptible strains and the effect size of GA in CNS disease require further study. Another recent study also called into question the effect of GA on the outcome of ischemic stroke. The publication by Kraft el al, also used a murine model (C57Bl/6) of 60 min tMCAo[15]. This study concluded that GA failed to protect from acute ischemic stroke. There were however, several limitations. Authors employed a pretreatment administration schedule that makes it clinically unfeasible. The presentation of GA was also Copaxone (Teva Pharmaceuticals) but was applied intravenously as opposed to the conventional subcutaneous route. Antigen presentation would be hindered using this administration scheme. Also the follow-up period was 24 hrs, insufficient time for the 3–4 days that are required for the adaptive immune response to become activated. The dose was different to the one used in our previous study and Poittevin et al. in this case they applied 3.5 mg/kg. The previous also employed the EAE-susceptible mouse strain C57Bl/6 in comparison to our studies that use an outbred EAE-resistant rat strain. The broad heterogeneity between methodologies makes it very difficult to compare these studies. One thing prevails, efforts should be made to standardize the optimal therapeutic dose, the most effective and safe adjuvant, and define the implication of the use of EAE-resistant *vs*. EAE-susceptible strains in PA research. Also we recommend that studies wishing to evaluate neurological recovery in acute ischemic stroke prolong their follow-up periods to more than 7 days.

There were some limitations to our study that will be modified during the next phase as we move towards the preclinical validation of Cop-1/GA for acute ischemic stroke. Future studies will evaluate the optimal time window for Cop-1/GA treatment after stroke. For ischemia-reperfusion models it is suggested that treatment be administered between the 90 and 180-min mark after MCAo to better represent the clinical therapeutic window of tPA. Although, therapies with a similar action pathway as Cop-1 have demonstrated to be useful up to 72 hrs after neurotrauma; therefore, Cop-1/GA may also benefit from administration times beyond 180 min[29]. Even though the results obtained by conventional counting demonstrated clear evidence of increased neurogenesis, the ideal method would be to use unbiased stereological counting. It is also important to determine if treatment with Cop-1/GA does not cause adverse side effects. Our study only observed injection site hypersensitivity in 8.6% animals. Another potential adverse side effect would be epilepsy due to an aberrant neurogenic response [30]. During the two-month follow-up we did not observe any clinical signs of seizures in any of our animals but this is not an objective method of evaluation. We suggest that future studies employ long-term cortical EEG recordings and other behavioral tasks to objectively assess whether Cop-1/GA-induced neurogenesis causes seizures, epilepsy or cognitive deficit.

## Conclusion

This study demonstrates that immunization with Cop-1 significantly improves neurological deficit and infarct volume after 60 days in a tMCAo rat model. This benefit is most likely mediated by enhanced neurogenesis at 7 and 60 days post-stroke with evidence that the consequent increase in NPCs results in new formation neurons in the ipsilateral CC. Neurotrophin analysis in the acute phase of stroke suggest that this process is most likely mediated by NT-3 and not by BDNF as previously thought for GA therapy in EAE. The present manuscript pushes the off-label use of GA for acute ischemic stroke closer to the clinical setting. Nonetheless, it also extols the need for studies analyzing optimal dosing, adjuvant safety and effectiveness, generalized neurological evaluations and an increase in follow-up periods for acute ischemic stroke studies.

## Supporting Information

S1 ARRIVE Guidelines Checklist (PDF)
